# Stiffening Effect of the [Bmim][Cl] Ionic Liquid on the Bending Dynamics of DMPC Lipid Vesicles

**DOI:** 10.1021/acs.jpcb.1c01347

**Published:** 2021-06-25

**Authors:** Pallavi Kumari, Antonio Faraone, Elizabeth G. Kelley, Antonio Benedetto

**Affiliations:** † Department of Sciences, University of Roma Tre, 00146 Rome, Italy; ‡ School of Physics and Conway Institute of Biomolecular and Biomedical Research, University College Dublin, Dublin 4, Ireland; § NIST Center for Neutron Research, National Institute of Standards and Technology, Gaithersburg, Maryland 20899, United States; ∥ Laboratory for Neutron Scattering, Paul Scherrer Institute, 5232 Villigen, Switzerland

## Abstract

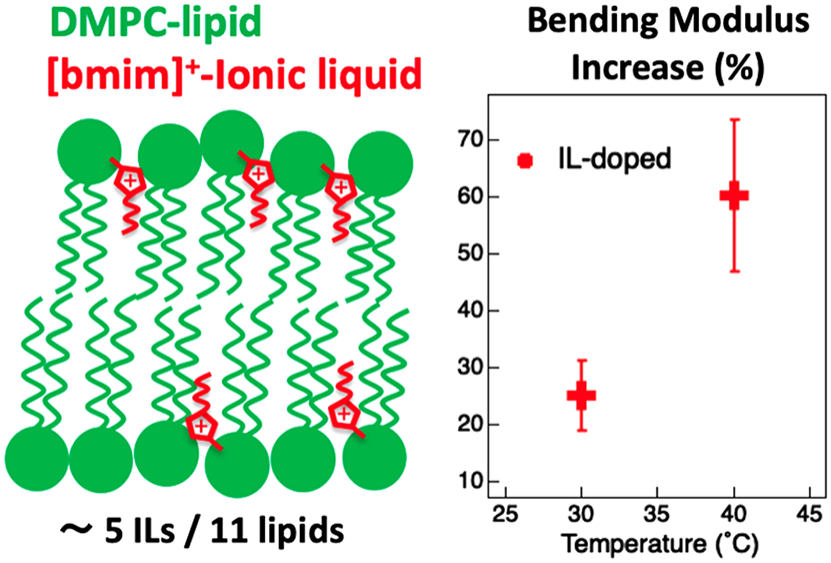

The elastic properties of the cellular lipid membrane play a crucial role for life. Their alteration can lead to cell malfunction, and in turn, being able to control them holds the promise of effective therapeutic and diagnostic approaches. In this context, due to their proven strong interaction with lipid bilayers, ionic liquids (ILs)—a vast class of organic electrolytes—may play an important role. This work focuses on the effect of the model imidazolium-IL [bmim][Cl] on the bending modulus of DMPC lipid vesicles, a basic model of cellular lipid membranes. Here, by combining small-angle neutron scattering and neutron spin–echo spectroscopy, we show that the IL, dispersed at low concentrations at the bilayer–water interface, (i) diffuses into the lipid region, accounting for five IL-cations for every 11 lipids, and (ii) causes an increase of the lipid bilayer bending modulus, up to 60% compared to the neat lipid bilayer at 40 °C.

## Introduction

It is well-accepted, nowadays, that cell membranes are not just simple physical barriers that separate the cell interior from the surrounding environment but that they play an active and crucial role for life.^1−3^ The biochemical function of cell membranes is carried out by a variety of different biomolecules, including a huge multitude of different lipids.^4−8^ Lipids account for about 50% of overall cell membrane composition. They are arranged in a single bilayer structure, whose viscoelasticity plays a major role in regulating the cell membrane biochemical function.^9−14^ Alteration in membrane viscoelasticity, consequently, can lead to cell malfunction. Along with membrane viscoelasticity, however, there are two other key characters into play here: (i) the cell stiffness, mainly associated with the cytoskeleton stiffness, and (ii) the cell matrix stiffness, both of which have been observed altered in several pathological conditions, including vascular disease, cancer, virus, and bacterial infections.^15−21^ Even though, it is complicated to disentangle the effects of each of these three viscoelastic contributions and clarify their cause–effect relationships, it is accepted that they all play a role in diseases. For example, cancer-related changes in the mechano-elastical properties of cells were previously thought to be limited to matrix stiffening.^16^ Later it has been shown that a significant softening of tumor cells is also taking place^21^ and, more recently, that membrane stiffness alone is sufficient to inhibit invasiveness of cancer cells.^22^ Thus, being able to control cell membrane viscoelasticity holds the promise of effective therapeutic and diagnostic approaches for treating diseases.^22,23^ In this context, due to their high lipophilicity and strong interaction with cell membranes, ionic liquids (ILs)—a relatively new and vast family of organic electrolytes—may play an important role.^24−26^

ILs are ionic compounds composed by an organic cation and either an organic or inorganic anion, which possess a variety of intriguing characteristics such as being liquid around room temperature.^27,28^ An initial set of biological studies^29−32^ have stimulated a more recent series of chemical-physical investigations on the interaction between ILs and biomolecules.^33,34^ Among those studies, a special focus has been dedicated to the investigation of IL-biomembrane interactions.^24−26,35−46^ In this context, neutron reflectivity^47^ and molecular dynamics (MD) simulations^48^ have shown the microscopic mechanism of insertion of imidazolium and choline IL-cations into phospholipid bilayers which, at equilibrium, results in about three to seven IL-cations for every ten lipids absorbed in the lipid phase. The MD simulations, moreover, have also shown that the presence of IL-cations in the lipid region does alter the bilayer bending modulus. The aim of this work is to explore this computational forecast experimentally. To do so, neutron spin–echo (NSE) spectroscopy has been employed to investigate the effect of one of the most studied ILs, i.e., [bmim][Cl] (1-butyl-3-methylimidazolium chloride), on the bending modulus of a model cellular lipid membrane represented by DMPC (1,2-dimyristoyl-*sn*-glycero-3-phosphocholine) lipid vesicles. DMPC has been chosen among other lipids and lipid mixtures because (i) PC-based lipids are the most abundant lipid-type in cell membranes and (ii) among the other PC-based lipids, DMPC is fluid at physiological temperatures.

This NSE study is supported by dynamic light scattering (DLS) and small-angle neutron scattering (SANS) investigations focused, respectively, on vesicle structural stability and IL-partitioning in IL-doped vesicles. In the following, details on sample preparation, methodology, and data analysis will be presented in a dedicated Materials and Methods section. Then, in the Results and Discussion, DLS and SANS results will be presented first, followed by the NSE study. To facilitate the reading of this work, a few figures are reported in the Supporting Information.

## Materials and Methods

### Materials

Fully protonated (h-DMPC) and tail-deuterated (d54-DMPC) 1,2-dimyristoyl-*sn*-glycero-3-phosphocholine (DMPC) lipid powders were purchased from Avanti Polar Lipids (Alabaster, AL) and used without further purification. Sample preparation and experiments were carried out above the gel-to-fluid main transition temperature of this lipid that is *T*_M_ = 24 °C for h-DMPC and *T*_M_ = 20 °C for d54-DMPC. The 1-butyl-3-methyl imidazolium chloride ([bmim][Cl]) ionic liquid was purchased from IoLiTec, Germany, and used without further purification. D_2_O 99.9% was purchased from Cambridge Isotope Laboratories (Andover, MA, USA).

### Preparation of DMPC Unilamellar Vesicles

Because replacing hydrogen atoms with deuterium atoms dramatically alters the neutron scattering length density contrast without significantly modifying the physicochemical properties of the system, combinations of protiated and deuterated chemicals were chosen to get appropriate samples for each (neutron scattering) measurement. More specifically, four different types of DMPC unilamellar vesicle samples were prepared: (i) h-DMPC in H_2_O (Milli-Q) for DLS, (ii) d54-DMPC in D_2_O, and (iii) d54-DMPC:h-DMPC (9:1 molar ratio) in D_2_O for SANS and (iv) h-DMPC in D_2_O for NSE. DMPC powder(s) was/were (co)-dissolved in chloroform (20 mg/mL) and dried under nitrogen gas flow and then under vacuum for 1 h. The necessary amount of D_2_O was added to the dried lipids to get the concentration of 100 mg/mL. DMPC unilamellar vesicles were prepared by extruding the lipid water suspension through a heated mini-extruder (Avanti Polar Lipids) with a porous polycarbonate membrane (pore diameter 100 nm) for 21 times at 60 °C. A series of these DMPC unilamellar vesicles were used as “neat samples”. For the “IL-doped samples”, appropriate amounts of [bmim][Cl] were added to the neat DMPC unilamellar vesicles to reach 0.1 mol/L (hereafter indicated by M) IL-concentration and wait for 1 h before use. For DLS and SANS measurements, the samples were further diluted to 1 mg/mL. For DLS, a higher IL-concentration of 0.5 M has been used. The vesicle solutions were kept at 60 °C until the measurements. Previous studies have shown that, at this low IL-concentrations, [bmim]-based ILs reduce the gel-to-fluid main phase transition temperature of the lipid by 1 to 2 deg only.^44,45^

### Dynamic Light Scattering

The vesicle size distributions were characterized on a DLS Zetasizer Nano-ZS device (Malvern Instruments) equipped with a He–Ne laser (λ = 633 nm). For the measurements, 10 mm diameter polystyrene semi-micro cuvettes were used as sample containers into which the DMPC unilamellar vesicles solutions were placed. The DLS method yields the normalized intensity time self-correlation function that was converted to relaxation time by an inverse Laplace transformation. The diffusion coefficient was obtained from the moments of the time distribution of the intensity, and the hydrodynamic diameter of the vesicles, *d*_*H*_, was finally obtained using the Stokes–Einstein equation:
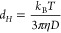
1where *D* is the diffusion coefficient, *k*_*B*_ is the Boltzmann constant, *T* is the absolute temperature, and η is the solvent (water in our case) viscosity. Experiments were carried out at the fixed scattering angle of 173 deg and at a temperature of 25 °C. Experiments were carried out in triplicates for both neat and IL-doped conditions, and mean values were computed for each of these independent experiments. Then, for each condition, we have used the associated 3 mean value diameters to compute the vesicle diameter (as their average) and the associated standard deviation, which have been retained as the DLS final result.

### Small-Angle Neutron Scattering

SANS measurements were performed at the NGB 30m SANS instrument at the NIST (National Institute of Standards and Technology) Center for Neutron Research (NCNR). The experiments were carried out at an incoming neutron wavelength of 6 Å (wavelength spread of 13.8%), and data were collected with a two-dimensional detector at three different sample-to-detector distances (1, 4, and 13 m) in order to cover the scattering vector range of 0.002 < *Q* (Å^–1^) < 0.4, where  with λ and θ as the incident neutron wavelength and scattering angle, respectively. Neat and IL-doped DMPC unilamellar vesicles samples were contained in 1 mm path-length quartz cells and were measured at two temperatures above the gel-to-fluid main transition temperature of the lipids, i.e., at 30 and 40 °C. The temperature was controlled with a recirculation bath with an accuracy of 0.1 °C. Data reduction was performed using the Igor Pro based reduction macros supplied by NIST to correct for detector sensitivity, instrumental background, empty cell, sample transmission, and solvent background, providing 1D *I*(*Q*) vs *Q* SANS profiles.^49^ Data analysis was performed with the software SasView.^50^ The unilamellar lipid vesicles were modeled by a polydisperse core 3-shell model composed of (i) a D_2_O polydisperse core, (ii) an inner lipid head layer, (iii) a lipid-tails double-layer, and (iv) an outer lipid head layer exposed to the solvent. The scattering intensity from dilute vesicles using a poly core three-shell model is given by^51^
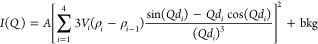
2where *A* is the scaling factor, bkg is the constant background, and *V*_*i*_, *d*_*i*_, and *ρ*_*i*_ are the volume, thickness, and scattering length density (SLD) of the core and the three shells, respectively. The SLDs of each molecular species, along with few other parameters, are reported in Table 1. The lipid mix d54-DMPC:h-DMPC (9:1 molar ratio) and pure d54-DMPC were used for the neat and IL-doped cases, respectively. d54-DMPC provides a significant scattering contrast between (i) headgroups and acyl chains, (ii) headgroup and D_2_O, and (iii) acyl chains and ILs, providing the best spatial resolution condition to resolve the lipid vesicles’ 3-layer structure (i.e., outer heads–2 tails–inner heads) and measure the IL-partitioning. For the first fitting iteration, the thicknesses of outer and inner head-regions and tail-region have been fixed to the values obtained by us by means of neutron reflectivity on a supported neat DMPC–lipid bilayer.^47^ For the second fitting iteration, those values have been allowed to relax slightly. For the IL-doped cases, these thicknesses as well as the radius of the polydisperse core have been fixed to the neat-case values; this is because the SANS data are not sensitive to the IL-induced 1 Å variations measured by neutron reflectometry.^47^

**Table 1:** Theoretical Scattering Length Densities (SLD) Calculated from the Neutron Scattering Length, *b*, of Each Atom and the Volume of Each Molecule^a^

material	formula	MW (g/mol)	volume (Å^3^)	density (g/cm^3^)	*b* (10^–4^ Å)	SLD (10^–6^ Å^–2^)
phospholipids						
h-DMPC	C_36_H_72_NO_8_P	677.945	1101	1.0225	3.1	0.282
h-DMPC, tails	C_26_H_54_	366.718	754	0.8076	–2.91	–0.386
h-DMPC (d54-DMPC), head	C_10_H_18_NO_8_P	311.227	347	1.4894	6.01	1.732
d54-DMPC	C_36_D_54_H_18_NO_8_P	732.269	1101	1.1044	59.31	5.387
d54-DMPC, tails	C_26_D_54_	421.042	754	0.9273	53.3	7.069
d54-DMPC:h-DMPC	n/a	n/a	n/a	n/a	n/a	4.85
d54-DMPC:h-DMPC, head	n/a	n/a	n/a	n/a	n/a	1.732
d54-DMPC:h-DMPC, tails	n/a	n/a	n/a	n/a	n/a	6.287
ionic liquid and water						
heavy water	D_2_O	20	30	1.107	1.9	6.35
[Bmim][Cl]	C_8_H_15_N_2_Cl	174.67	268.57	1.086	2.538	0.951
[Bmim]^+^	C_8_H_15_N_2_	139.27	167	1.38	1.580	0.94

a Chemical formula, molecular weights, and densities are also reported. d54-DMPC is the deuterated-tails version of h-DMPC. As a result, for both lipid versions, the heads are fully hydrogenated. The d54-DMPC:h-DMPC lipid mix has been prepared with a ratio of 9:1 of the two lipids, respectively.

### Neutron Spin–Echo

By accessing a time-scale of 0.01 to 100 ns and a length-scale of 1 to 10 nm, NSE is an ideal technique to probe thermal equilibrium undulation dynamics of cellular lipid membranes, which can then be modeled to access their elastic and viscous properties. Fully protonated lipids (h-DMPC) and D_2_O were used in NSE experiments to enhance the scattering signal from the vesicle’s lipid bilayer. NSE experiments on both neat and [bmim][Cl]-doped h-DMPC unilamellar vesicles were carried out at the physiological temperatures of 30 and 40 °C, in which the lipid bilayer is in the fluid phase, using the NG-A NSE spectrometer at the Center for High Resolution Neutron Scattering (CHRNS) at NCNR, Gaithersburg, MD. Incident neutron wavelengths of 8.0 or 11 Å were selected by a velocity selector (wavelength resolution of about 18%) to access a Q-range of 0.04 Å^–1^ to 0.11 Å^–1^ and Fourier times ranging from 0.01 to 100 ns corresponding to the bilayer bending undulations. Titanium NCNR-standard sample cells with quartz windows with a path length of 2 mm were used to load the samples. The temperature was controlled with a circulating bath with an accuracy of 0.1 °C, and the samples were equilibrated for 30 min before starting the measurements. The raw data were reduced and corrected for instrument resolution and D_2_O solvent background by the DAVE software package^52^ to obtain the normalized intermediate scattering function *I*(*Q,t*)/*I*(*Q*,0), where *Q* and *t* correspond to wavenumber transfer and Fourier time, respectively. Data analysis was performed with the software Mathematica. The accessible NSE *Q*-range corresponds to length scales that are smaller than the radius of the vesicles (*R* = 50 nm). In this circumstance, i.e., *QR* ≫ 1 for all measured *Qs*, the measured intermediate scattering function *I*(*Q,t*) can be modeled by the Zilman–Granek theory,^53^ based on Helfrich’s thin elastic membrane sheet model for bending energy, which describes thermal equilibrium single membrane fluctuation/undulation dynamics, i.e., bending motions, as follows:
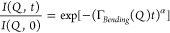
3in which *Γ*_*Bending*_ (*Q*) is the relaxation rate for bending fluctuation and α is the stretched exponent that corresponds to
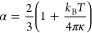
4For lipid bilayers, that is the case of the present study, κ/*k*_*B*_*T* is expected to be on the order of 10 or greater. As a result, the stretched exponent α can be approximated to 2/3. To double check this, the measured NSE-*I*(*Q*,*t*) profiles have been fitted with eq 3; the obtained values of α as a function of *Q* are shown in Figure S1 for all the systems and conditions considered in the present study. Overall, the condition α = 2/3 is satisfied, even though in the low-Q region α clearly deviates from 2/3 (this deviation could originate from the experimental resolution limit and/or the violation of the Zilman and Granek theory in the long-time region, e.g., due to the vesicles’ self-diffusion). As a result, the normalized *I*(*Q,t*)s can then be modeled as follows:
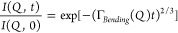
5in which the relaxation rate for bending fluctuations Γ_*Bending*_ (*Q*) is given by the expression
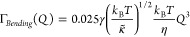
6where κ̃ is the “effective” bending modulus, η is the solvent viscosity, *k*_*B*_ is the Boltzmann constant, *T* is the temperature, and γ accounts for the orientational averaging between membrane domains and scattered neutrons. When *κ̃/k*_*B*_*T* ≫ 1, as in the present cases, γ can be set to 1. Accounting for the intermonolayer frictions, Watson and Brown^54^ showed that the effective bending modulus measured in NSE can be related to the “intrinsic” bending modulus, κ, by *κ̃ = c + 2h*^*2*^*k*_*m*_, in which *h* denotes the height of the neutral surface from the bilayer midplane and *k*_*m*_ = 12*κ*_*m*_/*h*_*c*_^2^ is the monolayer area compressibility modulus, where *κ*_*m*_ is the monolayer bending modulus and *h*_*c*_ is the monolayer hydrocarbon thickness. The monolayer parameters can be re-expressed in terms of the bilayer parameters as *κ*_*m*_ = *κ*/2, yielding κ̃ = {1 + 48 (*h*/2*h*_*c*_)^2^}κ. The height of the neutral surface cannot be measured experimentally and remains a topic of discussion in literature with values of *h*/2*h*_*c*_ ranging from 0.25 to 0.6. As suggested in a previous study on DMPC lipid vesicles,^55^*h*/2*h*_*c*_ = 0.5 was used here to analyze the data. Accepting these refinements, *Γ*_*Bending*_ (*Q*) can be rewritten as
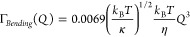
7In line with this theoretical framework, the bending moduli reported in this study have been obtained by fitting the measured NSE-*I*(*Q*,*t*) profiles with eq 5 to access *Γ*_*Bending*_ (*Q*), which have then been fitted with eq 7 to access the bending moduli, κ. Because of the low concentration of [bmim][Cl], for both neat and IL-doped samples, the viscosity of heavy water was used as the solvent viscosity, and the direct contribution of the IL to the measured signal was considered to be negligible.

In the literature, another fitting protocol to extract the bending modulus, κ, from NSE-*I*(*Q*,*t*) profiles has also been proposed and used during the years. We have also considered this approach for comparison purpose to check the consistency of our results. This alternative fitting protocol consists in merging all the *I*(*Q*,*t*) data of each sample and condition into an *I*(*t*·*Q*^3^) master data set and then fit it with eq 8 to access directly κ.^56^Equation 8 is the combination of eq 5 and eq 7.
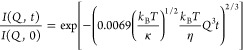
8Finally, it is worth mentioning that the thin elastic sheet model predicts a direct relationship between the bending modulus, κ, and the area compressibility modulus, *K*_*A*_:
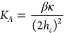
9where *h*_*c*_ is the monolayer hydrocarbon thickness and β is a constant depending on the coupling between the two lipid leaflets, with β = 12 for fully coupled and β = 48 completely decoupled monolayers. As suggested in a previous study,^57^ β = 24 could be used here to calculate *K*_*A*_ by combining *h*_*c*_ values measured using SANS and κ values from NSE. However, the presence of the IL in the lipid region could alter the coupling between the two bilayer leaflets. As a result, to have a realistic estimation of *K*_*A*_, the values of β should be accessed by an independent additional measurement, e.g., by probing the thickness fluctuations of neat and IL-doped bilayers.

## Results and Discussion

### Dynamic Light Scattering Study

**Figure 1:**
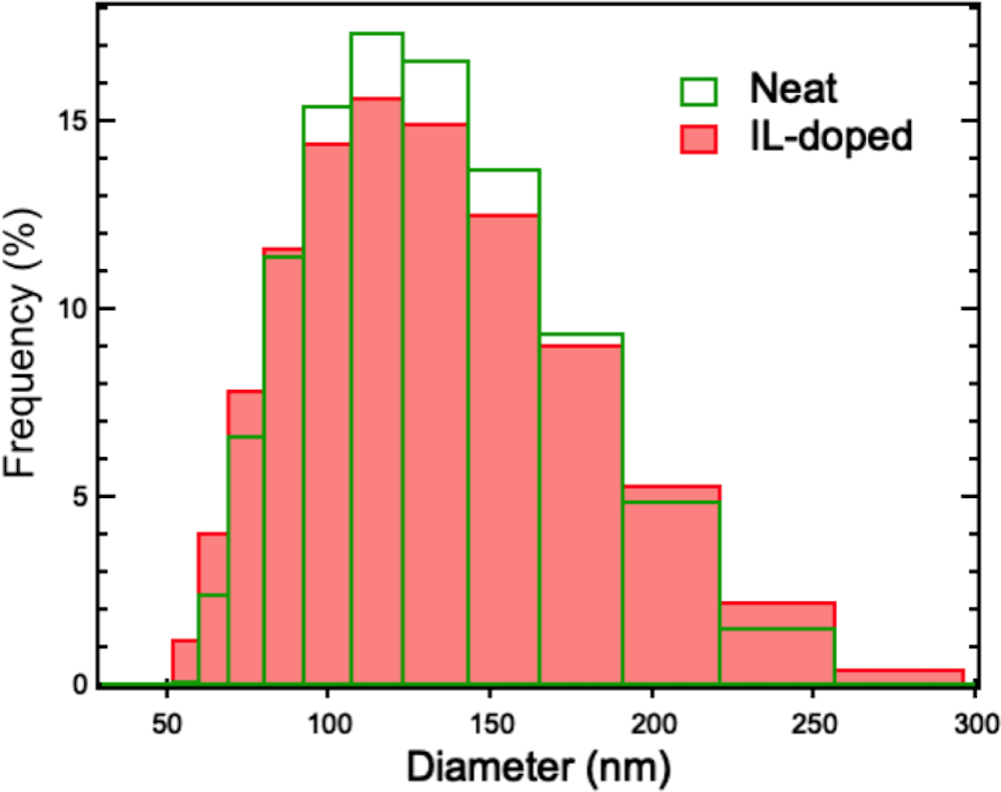
Representative diameter distribution functions for neat (green) and 0.5 M IL-doped (red) DMPC–lipid vesicles obtained by DLS at 25 °C, i.e., in the fluid phase.

DLS has been employed to check the structural integrity of the lipid vesicles upon the addition of the IL. Neat and 0.5 M [bmim][Cl]-doped 100 nm-diameter unilamellar DMPC lipid vesicles have been prepared in water at a 1 mg/mL concentration for the DLS measurements. The IL-concentration has been set quite below its critical micellar concentration (CMC = 5 M) to avoid any structural damage of the lipid vesicles.^35^ By measuring the time self-correlation function, the DLS measurements gave access to the (i) mean diffusion coefficient, *D*, and the (ii) hydrodynamic diameter, *d*_*H*_, of the lipid vesicles in both neat and IL-doped variants (eq 1). The diameter distributions for the neat and the IL-doped lipid vesicle systems were identical and overlapped even after a day of incubation in the IL (Figure 1). The average diameter and the mean diffusion coefficient resulted, in turn, to be the same for both cases. More specifically, the average diameter resulted to be (110 ± 2) nm; the mean diffusion coefficient resulted to be (0.56 ± 0.01) × 10^–4^ cm^2^/s and (0.70 ± 0.01) × 10^–4^ cm^2^/s at 30 and 40 °C, respectively, corresponding to a diffusion relaxation time, *τ*_*diffusion*_ = 1/*DQ*^2^, in the range of (0.1–1.4) × 10^–10^ s. In conclusion, the DLS results confirm that, at this relatively low IL-concentration of 0.5 M, [bmim][Cl] was not affecting either the overall stability of the lipid vesicles or their self-diffusion. The lower concentration of 0.1 M of [bmim][Cl] was then used in the NSE study to fully guarantee that the vesicles were structurally stable as well as to minimize the direct contribution of the IL to the measured relaxation dynamics and its effect on the vesicles’ self-diffusion.

### Small-Angle Neutron Scattering Study

**Figure 2:**
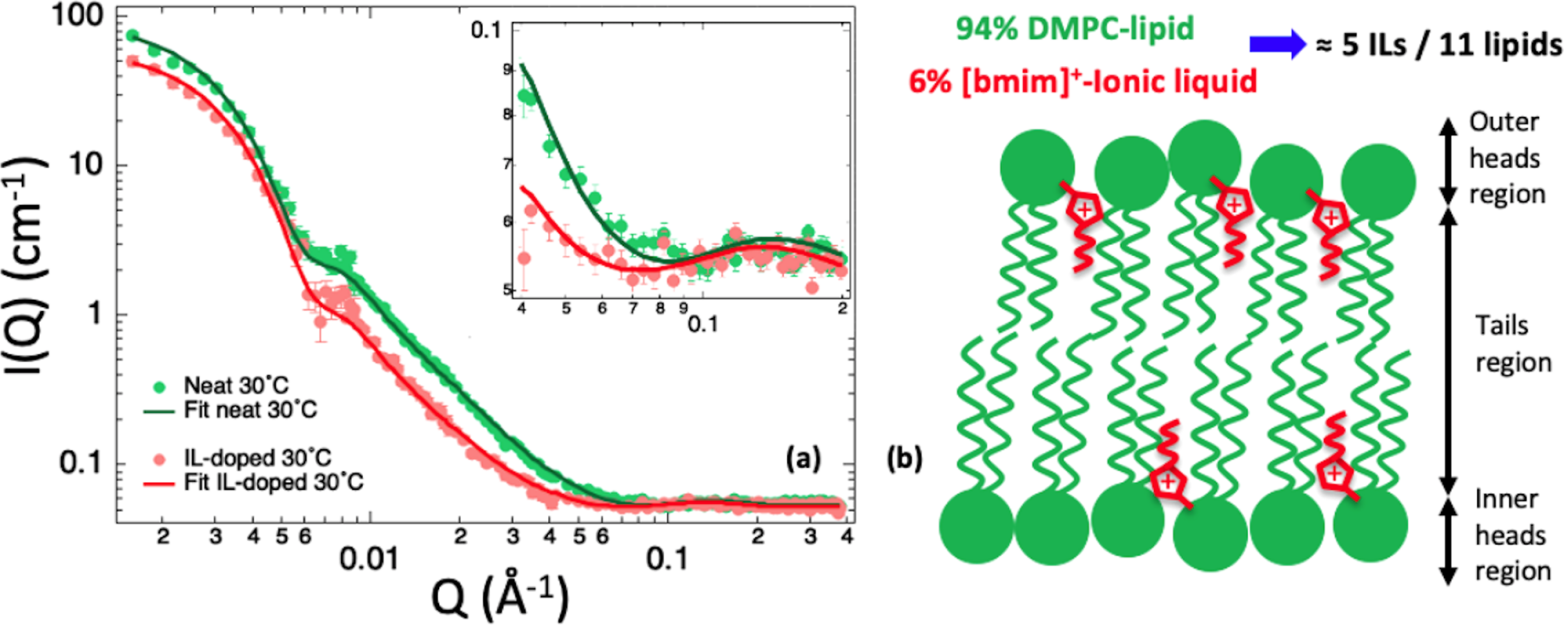
(a) SANS data (circles) collected on neat (green) and IL-doped (red) tail-deuterated DMPC–lipid vesicles at 30 °C together with the fitting curves obtained using the polydisperse core 3-shell model of eq 2. Fit parameters and results are reported in detail in Table 1 and Table 2, respectively. In the inset, the zoom-in in the 0.04–0.2 Å^–1^ Q-range region is presented. Error bars represent standard deviations. (b) Sketch, based on the SANS results, of the IL-doped lipid bilayer with DMPC–lipids in green and IL-cations in red: about 5 IL-cations per 11 lipids diffuse into the lipid region. The sketch is in scale with the lipid chain-length being about 3 times the IL chain-length.

SANS has been employed to study the partitioning of the IL (cation) between the water and lipid phases at the same two temperatures considered for the NSE study, which are 30 and 40 °C. Both temperatures are above the fluid-to-gel main phase transition of the DMPC lipid. Neat and 0.1 M [bmim][Cl]-doped 100 nm-diameter unilamellar DMPC lipid vesicles have been prepared in water at 1 mg/mL concentration for the SANS measurements. In this case, however, deuterated-tail lipids have been used to provide the best spatial resolution condition to resolve the vesicles inner structure and better compute the IL-cation partitioning. The experimental data have been modeled by a core 3-shell model composed of (i) a D_2_O polydisperse core, (ii) an inner lipid-heads layer, (iii) a lipid-tails double-layer, and (iv) an outer lipid-heads layer exposed to the solvent.^51^Figure 2a reports experimental data and fit curves for both neat and IL-doped DMPC vesicles at 30 °C; the equivalent figure for the 40 °C case is Figure S2. The complete results of the fits are reported in Table 2. By comparing the neutron SLD of the lipid-tail layer measured for the IL-doped sample with the neat case, the amount of IL-cations diffused into the lipid bilayer region was determined with good accuracy. The IL-cations account for (6.1 ± 0.4) % and (6.5 ± 0.4) % of the bilayer volume at 30 and 40 °C, respectively, corresponding to approximately five IL-cations per each 11 lipids (Figure 2b). Notably, this result is in good agreement with a previous investigation performed by neutron reflectivity on the partitioning of [bmim]^+^ cations into single supported lipid bilayers of POPC (1-palmitoyl-2-oleoyl-glycero-3-phosphocholine) and DMPC,^47^ and with full-atom molecular dynamics simulations,^48^ suggesting, moreover, that SANS could be routinely used for IL-partitioning studies in lipid vesicles.

**Table 2:** Structural Parameters Obtained by Fitting the SANS Data of Neat and IL-Doped DMPC Unilamellar Vesicles at 30 and 40 °C^a^

fit parameters	neat 30 °C	neat 40 °C	IL-doped 30 °C	IL-doped 40 °C
core radius, *R*_*core*_	437.7 (5.2)	440.5 (5.3)	437.7 (5.7 – fix)	440.5 (6.8 – fix)
core SLD, *SLD*_*core*_	6.3 (0.005 – fix)	6.3 (0.005 – fix)	6.3 (0.005 – fix)	6.3 (0.005– fix)
inner head layer thickness, *t*_1_	9.6 (1.5 – fix)	9.6 (2 – fix)	9.6 (1 – fix)	9.6 (1.5 – fix)
inner head layer SLD, SLD_1_	3.0 (0.8)	3.0 (0.8)	4.9 (0.4)	4.9 (0.6)
tails layer thickness, *t*_2_	29.0 (0.6 – fix)	29.0 (1 – fix)	29.0 (0.6 – fix)	29.0 (1.3 – fix)
tails layer SLD, SLD_2_	6.29 (0.05)	6.29 (0.05)	6.54 (0.03)	6.5 (0.03)
outer head layer thickness, *t*_3_	9.6 (1.5 – fix)	9.6 (2 – fix)	9.6 (1 – fix)	9.6 (1.5 – fix)
outer head layer SLD, SLD_3_	3.4 (0.9)	3.4 (0.9)	5.1 (0.4)	4.9 (0.5)
solvent SLD, SLD_*solvent*_	6.37 (0.001 – fix)	6.37 (0.001 – fix)	6.37 (0.001 – fix)	6.37 (0.001 – fix)
χ^2^	1.7	1.4	1.5	1.5

a The uncertainties, reported in parentheses, are standard deviations. Where “fix” is present, it means that the first fitting iteration has been done by fixing that value to either the neutron reflectivity value or the neat value for, respectively, neat and IL-doped cases.^47^ All the lengths are in Å, and the SLD is in 10^–6^ Å^–2^. The core radius poly-dispersity ratio is 0.3 for all the fits.

### Neutron Spin–Echo Study

**Figure 3:**
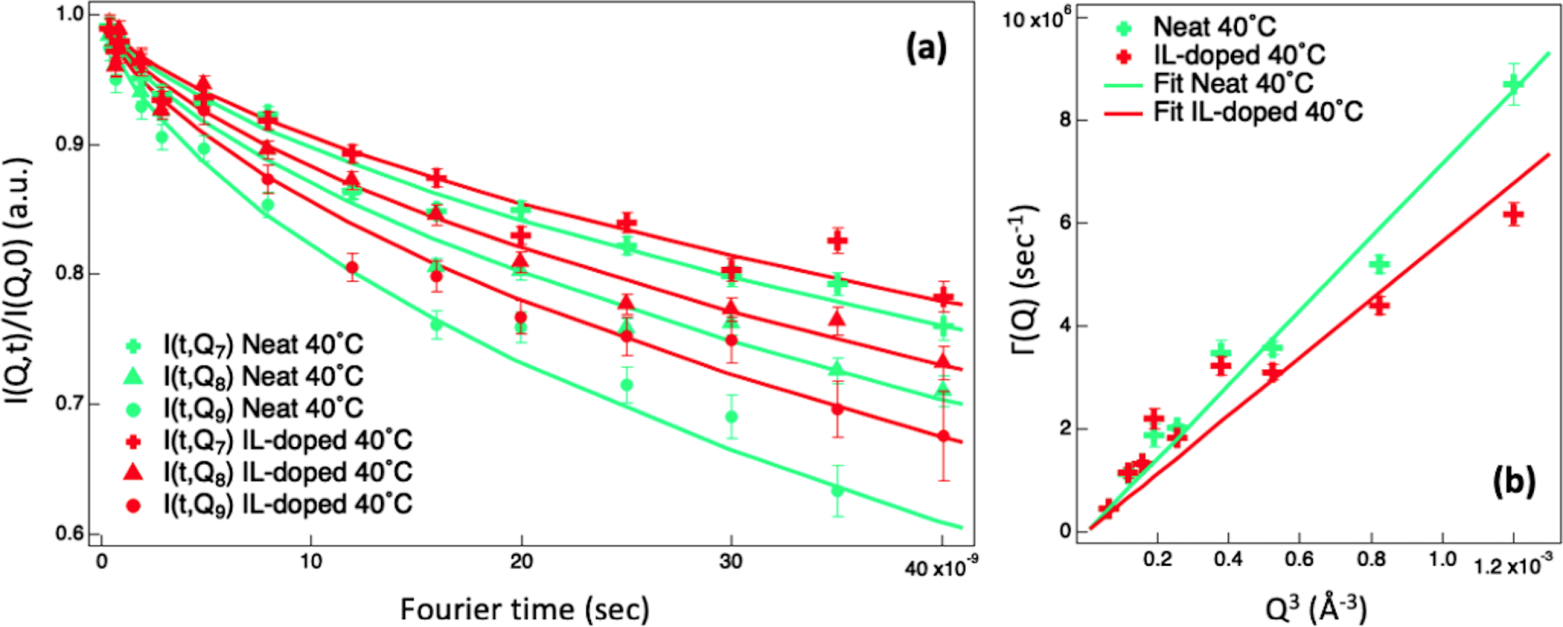
(a) Normalized intermediate scattering functions measured by NSE at 40 °C along with eq 5 fitting curves for neat (green) and [bmim][Cl]-doped (red) protonated DMPC–lipid vesicles in D_2_O at *Q*_7_ = 0.081 Å^–1^, *Q*_8_ = 0.094 Å^–1^, and *Q*_9_ = 0.106 Å^–1^. (b) Relaxation rate for bending fluctuations, *Γ*_*Bending*_(*Q*), versus *Q*^3^ together with eq 7 fitting curves. Error bars represent standard deviations. The equivalent figure for the 30 °C case is shown in Figure S3.

With the absence of structural damage confirmed by DLS and the quantification of the IL-partitioning into the lipid phase accessed by SANS, the effect of the absorbed [bmim]^+^ IL-cations on the membrane elasticity has been investigated by NSE. By encoding the system relaxation dynamics in the neutron spin precession, NSE is able to experimentally access a very extended time-window, usually covering from a fraction up to few hundreds of nanoseconds.^58^ Taken together with the ability of neutrons to distinguish between hydrogen and deuterium atoms,^58−60^ this extended accessible time-window allows NSE to probe bending and thickness fluctuations of model biomembranes, which are nano-to-microsecond membrane thermal equilibrium motions.^58,61^ Although NSE has been already used successfully a good number of times to compute the bending modulus of lipid bilayers, including DMPC–lipid bilayers,^61−69^ this is, to the best of our knowledge, the first NSE study on the effect of an IL on the elasticity of a lipid bilayer. To maximize the neutron scattering signal coming from the membrane bending dynamics, as common practice in those NSE investigations, fully hydrogenated DMPC lipids were dispersed at high concentrations in D_2_O rather than in H_2_O. Consequently, 100 mg/mL D_2_O solutions of neat and 0.1 M [bmim][Cl]-doped 100-nm-diameter unilamellar DMPC lipid vesicles have been prepared for the NSE measurements. As a result, the measured NSE signal describes the thermal equilibrium collective membrane height fluctuation/undulation dynamics, i.e., bending motions, and can be modeled using the Zilman–Granek theory of eq 5.^53^ To investigate the temperature dependence of the bending elasticity, the measurements have been carried out at two different temperatures, 30 and 40 °C. Both temperatures are above the fluid-to-gel phase transition temperature of the used lipid (*T*_M_ = 24 °C) and within the physiological temperature range. As a result, the experimental conditions mimicked the standard physiological condition in which cellular lipid membranes are in the fluid phase and experience variations in temperature of a few degrees only. Figure 3a reports the reduced experimental data together with the eq 5 fitting curves for selected *Q*-values for both pure and IL-doped DMPC vesicles at 40 °C. The equivalent figure for the 30 °C case is Figure S3. By looking at Figure 3a, it is possible to immediately observe a measurable effect of the IL on the collective relaxation dynamics of the DMPC–lipid membrane. More specifically, the presence of the IL decreases the characteristic relaxation rate of the bending fluctuations of the lipid membrane in a *Q*-dependent manner. In addition to this collective bending motion, lipids experience individual motions as well. In this context, by means of quasi-elastic neutron scattering (QENS), it has been shown that whereas [bmim][BF_4_] does not alter lateral and internal lipid motions, the longer tail [dmim][BF_4_] does.^43−45^

**Figure 4:**
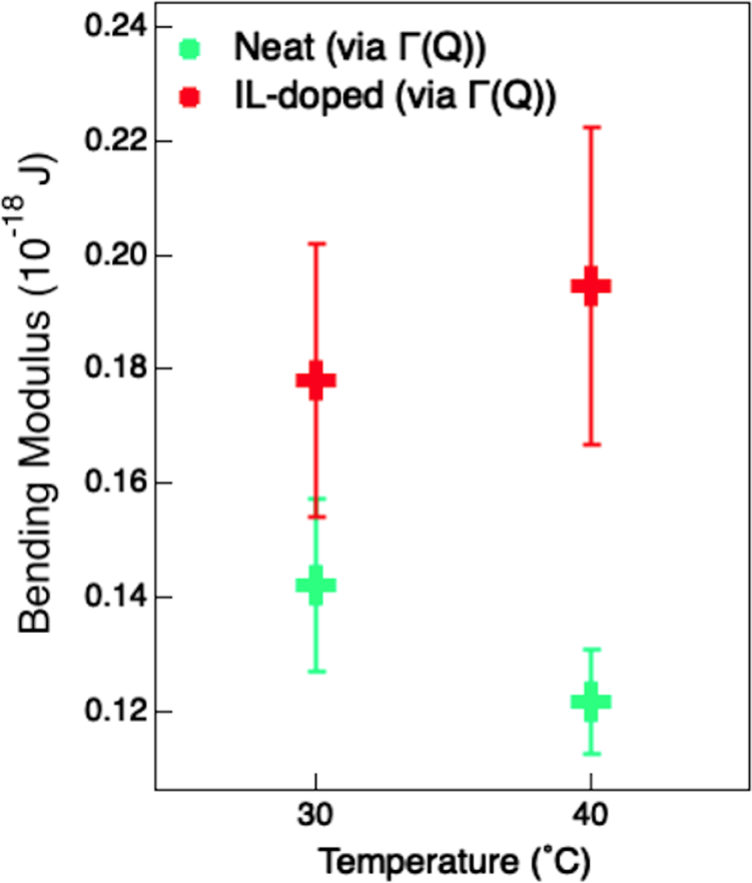
Bending modulus, κ, for neat (green) and [bmim][Cl]-doped (red) protonated DMPC–lipid vesicles in D_2_O at 30 and 40 °C obtained with eq 7. Error bars represent standard deviations.

In agreement with the predictions of the Zilman–Granek theory,^53^ the computed *Γ*_*Bending*_(*Q*) scales linearly with *Q*^3^ (Figure 3b). The bending moduli, κ, obtained by fitting *Γ*_*Bending*_(*Q*) with eq 7, are reported in Figure 4. They clearly show that the effect of the IL is to increase the bending modulus of the lipid vesicle and that this increment seems to increase with increasing temperature. Notably, the bending modulus values obtained for the neat case are in good agreement with the literature values,^55^ even though a small degree of multilamellarity has been observed (Figure S4). The eq 7 fits of Figure 3b capture the major features, with small deviations in the low-*Q* region pointing to the need to refine the basic Zilman–Granek model. For comparison, the NSE data have also been analyzed with the alternative *I*(*t*·*Q*^3^)-master fitting protocol of eq 8 mentioned in the Materials and Methods (Figure S5). The values of κ obtained with these two alternative fitting protocols are reported in Figure S6 and Table S1 for comparison. As can be seen, they are in good agreement one to the other: i.e., the differences in the values of κ, for a given condition, are within the associated error bars, meaning that the two fitting methods are basically providing the same κ values.

### Discussion

**Figure 5:**
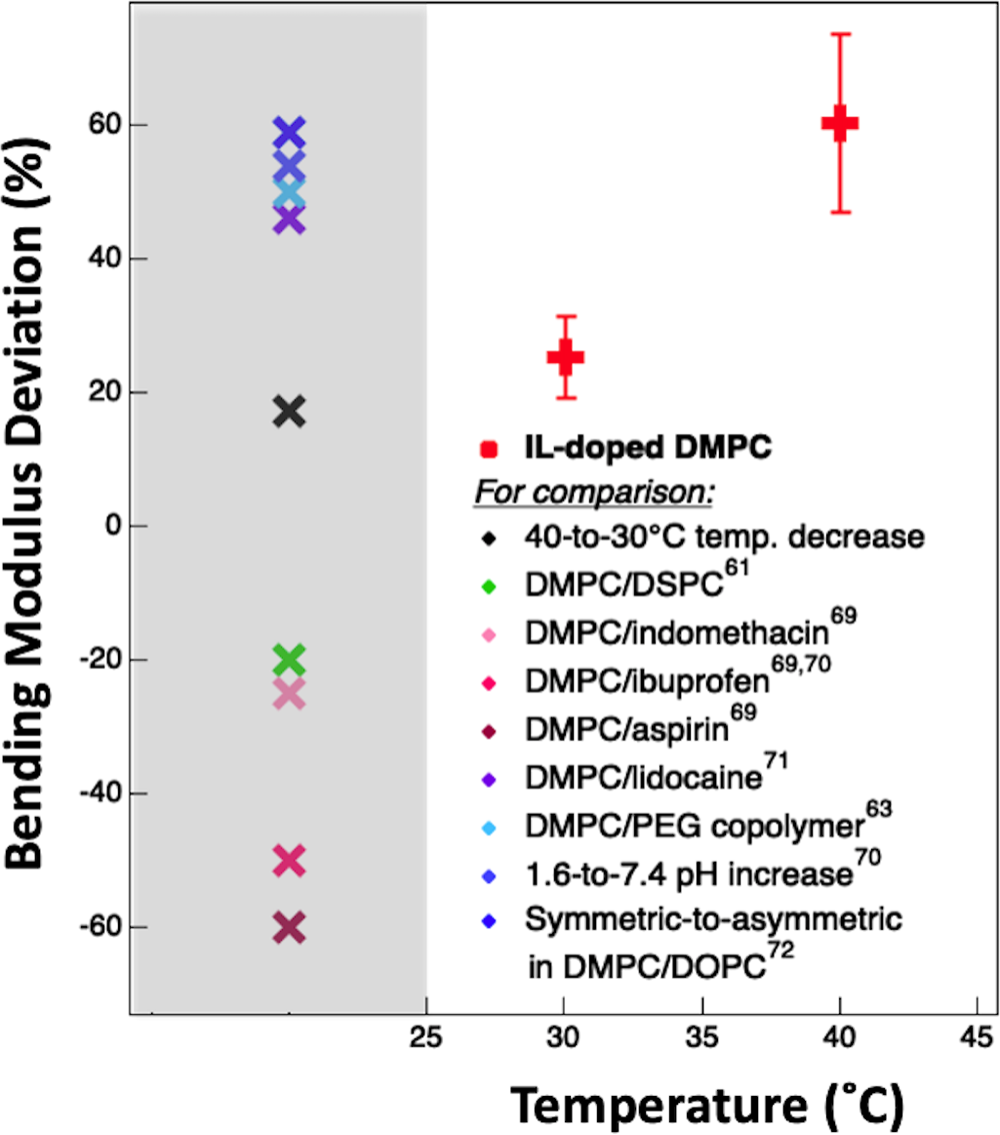
Bending modulus percentage deviation of IL-doped DMPC–lipid vesicles with respect to neat, 100(*κ*_*IL*_ – *κ*_*neat*_)/ *κ*_*neat*_, at 30 and 40 °C (red), showing the IL-induced increase in bilayer bending modulus. Error bars have been calculated by directly propagating the standard deviations in κ. To judge the extent and the physiological relevance of this IL-effect, several bending modulus data points extracted from the literature on DMPC (in the fluid phase and typically at 30 °C) are reported in a dedicated gray column on the left of the figure; see the main text for more details. Among those data, the “40-to-30 °C temperature decrease” (black) has been computed from our NSE data as the percentage deviation in the bending modulus of neat DMPC–lipid vesicles with decreasing temperature from 40 to 30 °C, i.e., 100(κ_*neat*_^30^°^C^ – κ_*neat*_^40^°^C^)/κ_*neat*_^40^°^C^.

Here, to facilitate the comparison with other literature data, the data are presented as the percentage increase between IL-doped and neat bending moduli, i.e., 100(*κ*_*IL*_ – *κ*_*neat*_)/*κ*_*neat*_ (Figure 5 and Figure S7). For both the temperatures considered in this study, the bending modulus of the IL-doped lipid membrane was higher than the neat membrane condition, corresponding to an increment of (25.2 ± 6.1)% and (60.4 ± 13.3)% at 30 and 40 °C, respectively. Notably, as far as the neat case is concerned, the increase in bending modulus with decreasing temperature from 40 to 30 °C is (17.1 ± 3.1)%. This value is reported in Figure 5 for comparison.

To better judge the extent of the IL-effect, the effect of other molecules on the bending modulus of DMPC–lipid bilayers is briefly considered here. It has been shown, for example, that the bending modulus of DMPC bilayers (i) reduces by 20% due to the addition of 0.2 to 0.6 mole fraction of DSPC lipid;^61^ (ii) reduces by 60%, 50% and 25% due to the addition of aspirin, ibuprofen, and indomethacin;^69,70^ (iii) increases by 46% due to the addition of lidocaine;^71^ (iv) increases by 59% due to the addition of the end-phosphorylated polyethylene glycol triblock copolymer;^73^ and (v) increases by 50% with increasing the pH value from 1.6 to 7.4.^70^ For all the listed cases, the bending modulus has been accessed by NSE spectroscopy, the bilayers were in the fluid phase, and only small variations of the order of an Ångstrom in the bilayer thickness have been recorded. Moreover, it has been also shown that (vi) moving from symmetric to asymmetric lipid distribution in DMPC/DOPC bilayers increases the bending modulus by 54%,^72^ (vii) the addition of cholesterol increases the bending modulus of DMPC bilayers in a concentration-dependent manner ranging from 20% up to 150%,^73^ and (viii) an ionic surfactant increases by 25% the bending modulus of a nonionic bilayer.^74^ These values have been reported on the left side of Figure 5 (in a dedicated *control column* in gray) for better comparison with the variations induced by the IL. This comparison suggests that the effect of the IL on the membrane bending modulus is in the range of other observed *physiologically relevant* cases.

## Conclusions and Remarks for the Future

To summarize, the effect of a well-known IL on the bending modulus of a model cellular lipid membrane has been investigated by NSE spectroscopy. The main scientific result here is that a subtoxic dose of the [bmim][Cl] IL can increase the membrane bending modulus of DMPC–lipid bilayer in a temperature-dependent manner, reaching (60.4 ± 13.3) % at 40 °C and that this increase is *physiologically relevant*. Table 3 summarizes the major results. DMPC lipids, however, are only one of the several lipid molecules that compose cell membranes. For instance, future studies should consider different lipid types as well explore the effect of IL concentration. An extra degree of freedom is also added by the huge variety of ILs, including ILs based on amino acids, choline, and phosphocholine, and ILs with magnetic properties, just to cite a few.^75−77^ As a result, the ability of an IL to act on the cellular membrane elasticity, as presented in this work, together with the huge variety of lipids and ILs combinations provides a new playground for research, which also holds the promise for novel applications in bionanomedicine and bionanotechnology including, for example, the use of ILs to control the release of drugs from drug-carrier liposomes, or to control stem cell differentiation and cell migration on substrates, or to develop new diagnostic tools for cancer detection, e.g. ref (25). In this bio-applied context, it is important to remember, however, that along with lipids several other different biomolecules are present in real biomembranes, including membrane proteins and saccharides, that are all surrounded by an electrically charged environment. As a result, future studies should also closely investigate the potential cross-interactions between ILs and these other cell membrane biomolecules.

**Table 3:** Summary of the Structural (SANS) and Elastic (NSE) Properties of Neat and [bmim][Cl]-Doped DMPC–Lipid Unilamellar Vesicles at 30 and 40 °C^a^

Temperature (°C)	Bilayer IL-volume fraction (%)	IL-number/11 lipids	IL-induced bending elasticity increase (%)
30	6.1 (0.4)	4.7 (0.3)	25.2 (6.1)
40	6.5 (0.4)	5.1 (0.3)	60.4 (13.3)

a The uncertainties are reported in brackets and represent 1 standard deviation. The complete set of structural SANS and elastic NSE results are reported in Table 2 and Table S1, respectively.

## Supporting Information Available

Stretched exponent α versus *Q* obtained by fitting the NSE experimental data with eq 3 (Figure S1), SANS profiles of neat and IL-doped d54-DMPC lipid vesicles at 40 °C together with their eq 2 fitting curves (Figure S2), NSE intermediate scattering functions and relaxation rates for bending fluctuations for neat and [bmim][Cl]-doped h-DMPC vesicles in D_2_O at 30 °C along with their eq 5 and eq 7 fitting curves (Figure S3), SANS profiles of neat and IL-doped h-DMPC lipid vesicles in D_2_O at 100 mg/mL concentration used in the NSE experiments (Figure S4), NSE intermediate scattering master-functions for protonated DMPC in D_2_O along with their eq 8 fitting curves (Figure S5), bending modulus for neat and [bmim][Cl]-doped h-DMPC lipid vesicles in D_2_O obtained with the two different fitting protocols described in the Materials and Methods (Figure S6), bending modulus for neat and [bmim][Cl]-doped h-DMPC lipid vesicles in D_2_O obtained with the two different fitting protocols described in the Materials and Methods (Table S1), and bending modulus percentage increases of IL-doped DMPC lipid vesicles with respect to neat obtained with the two fitting methods described in the Materials and Methods (Figure S7) (PDF)
