# Supplementary material for: Stiffening Effect of the [Bmim][Cl] Ionic Liquid on the Bending Dynamics of DMPC Lipid Vesicles
Source: J Phys Chem B. 2021 Jun 25;125(26):7241–50. doi: 10.1021/acs.jpcb.1c01347 (PMC8279542; doi:10.1021/acs.jpcb.1c01347)
Supplement: Supplementary file 1 — jp1c01347_si_001.pdf [file jp1c01347_si_001.pdf]

Stiffening Effect of the [Bmim][Cl] Ionic Liquid on the Bending Dynamics of DMPC Lipid Vesicles

Pallavi Kumari[1,2], Antonio Faraone[3], Elizabeth G. Kelley[3], and Antonio Benedetto[1,2,4,*]

[1]Department of Sciences, University of Roma Tre, 00146 Rome, Italy
[2]School of Physics, and Conway Institute of Biomolecular and Biomedical Research, University College Dublin, Dublin 4, Ireland
[3]NIST Center for Neutron Research, National Institute of Standards and Technology, Gaithersburg, Maryland 20899, USA
[4]Laboratory for Neutron Scattering, Paul Scherrer Institute, 5232 Villigen, Switzerland
[*]Corresponding author: antonio.benedetto@uniroma3.it antonio.benedetto@ucd.ie antonio.benedetto@psi.ch

Supporting Information

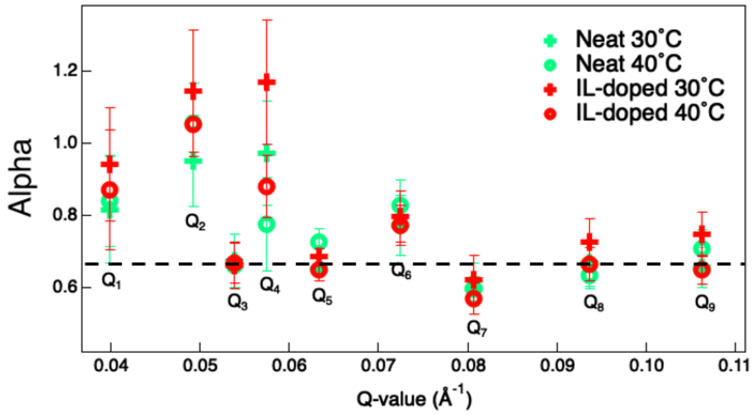

Figure S1 – Stretched exponent α versus Q, obtained by fitting the experimental data with eq. 3. The dashed horizontal line shows the value α=2/3. The Q-values are as follows: Q_1= 0.040 $Å^{-1}$; Q_2= 0.049 $Å^{-1}$; Q_3= 0.054 $Å^{-1}$; Q_4= 0.057 $Å^{-1}$; Q_5= 0.063 $Å^{-1}$; Q_6= 0.072 $Å^{-1}$; Q_7= 0.081 $Å^{-1}$; Q_8= 0.094 $Å^{-1}$; Q_9= 0.106 $Å^{-1}$. Error bars represent standard deviations.

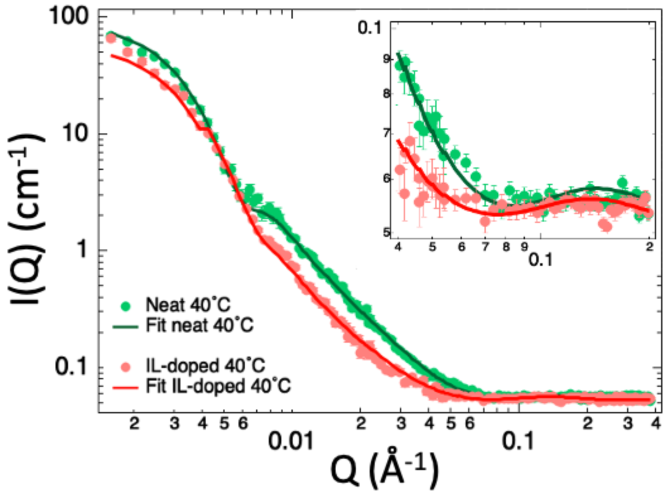

Figure S2 – SANS data (circles) collected on neat (green) and IL-doped (red) tail-deuterated DMPC-lipid vesicles at 40 ˚C together with the fitting curves obtained using the polydisperse core 3-shell model of eq. 2. Fit parameters and results are reported in detail in Table 1 and Table 2, respectively. In the inset, the zoom-in in the 0.04-0.2 $Å^{-1}$ Q-range region is presented. Error bars represent standard deviations.

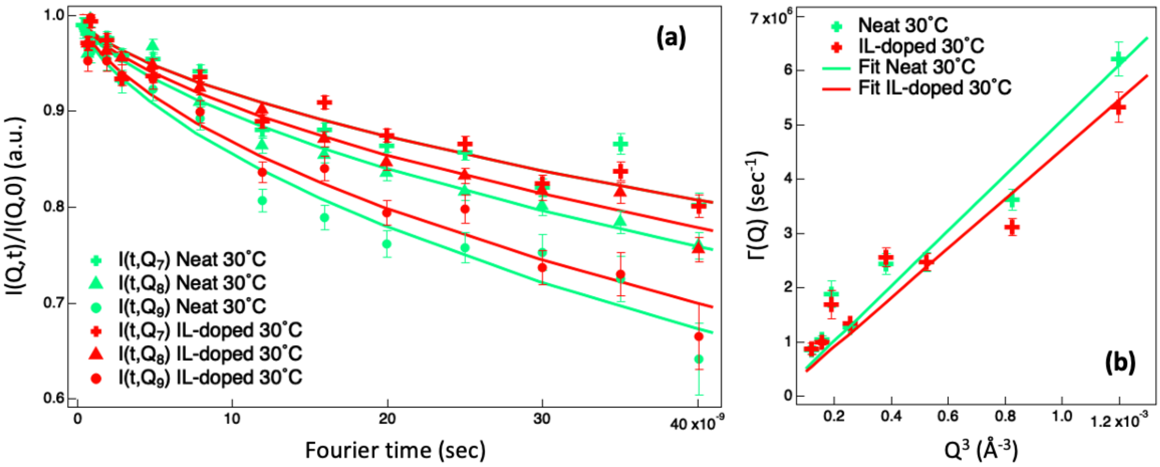

Figure S3 – (a) Normalized intermediate scattering functions measured by NSE for protonated DMPC in D_2O at 30˚C along with their eq. 5-fitting curves for neat (green) and [bmim][Cl]-doped (red) lipid vesicles at, from top to bottom, Q_7= 0.081 $Å^{-1}$, Q_8= 0.094 $Å^{-1}$, and Q_9= 0.106 $Å^{-1}$. (b) Relaxation rate for bending fluctuations, $\Gamma_{Bending}(Q)$, versus Q^3 together with eq. 7-fitting curves. Error bars represent standard deviations.

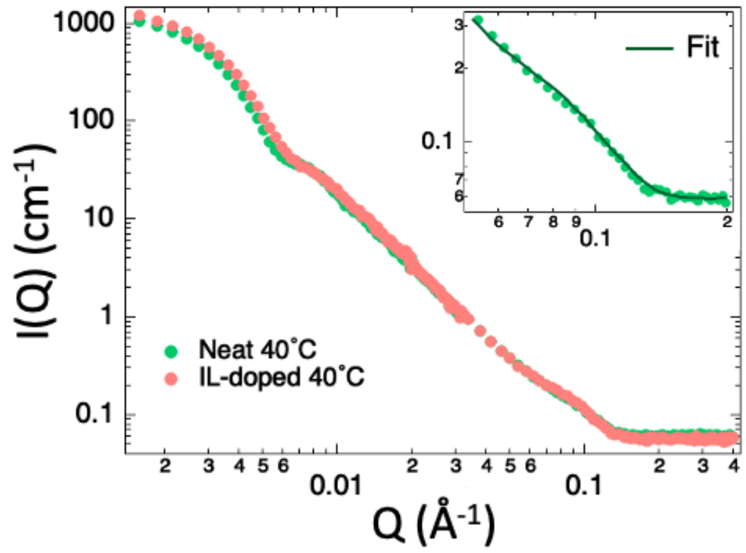

Figure S4 – SANS of the neat and IL-doped h-DMPC vesicles in D_2O used in the NSE experiments to measure the collective height fluctuations (100 mg/ml). The small shoulder in the data around Q ≈ 0.1 $Å^{-1}$ indicated the presence of a small population of multilamellar or paucilamellar vesicles in the samples. (Inset) Fitting the data with a multilamellar vesicle form factor suggests that the samples were > 90% unilamellar. While the presence of the small population of paucilamellar vesicles could affect the dynamics measured with NSE, the population was similar in both the neat and IL-doped samples, suggesting that we can reasonably compare the relative changes in the membrane stiffness.

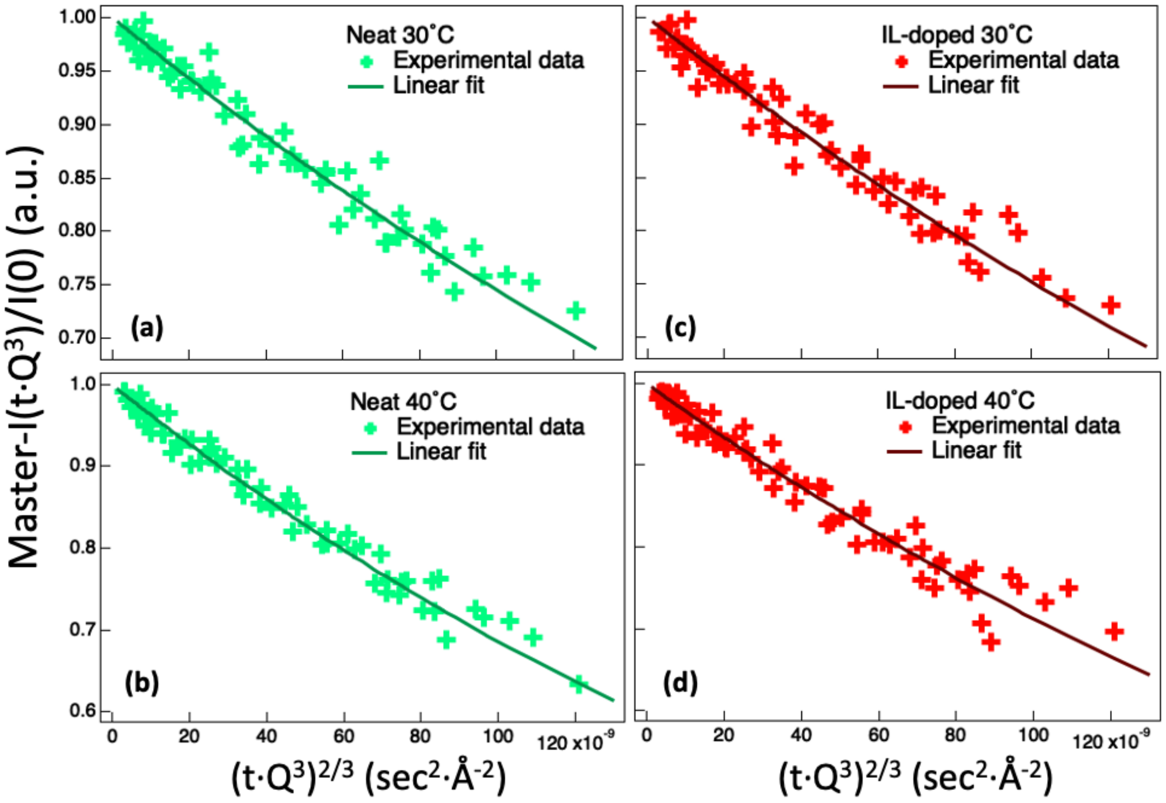

Figure S5 – Normalized intermediate scattering master-functions measured by NSE for protonated DMPC in D_2O along with their eq. 8-fitting curves for (a) neat 30 ˚C, (b) neat 40˚C, (c) [bmim][Cl]-doped 30˚C, and (d) [bmim][Cl]-doped 40 ˚C lipid vesicles.

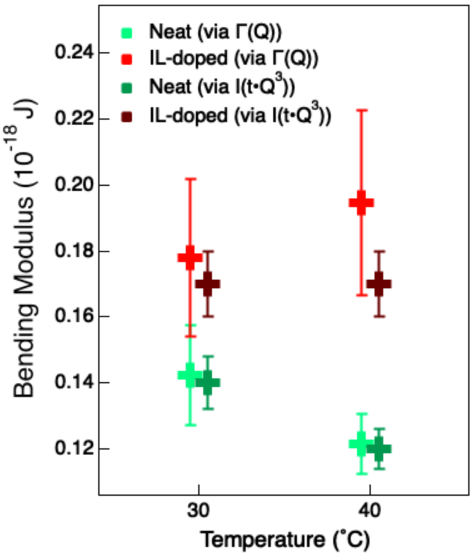

Figure S6 – Bending modulus, κ, for neat (greenish) and [bmim][Cl]-doped (reddish) protonated DMPC-lipid vesicles in D_2O at 30 ˚C and 40 ˚C obtained with the two different fitting protocols described in the *Materials and Methods* section. Error bars represent standard deviations.

	**Bending Modulus (10^{-18} J)** (via $\Gamma(Q)$, eq. 7)	**Bending Modulus (10^{-18} J)** (via I(t·Q^3)−mastercurve, eq. 8)
**Neat bilayer 30˚C**	0.14 (0.02)	0.14 (0.01)
**Neat bilayer 40˚C**	0.12 (0.01)	0.12 (0.01)
**IL-doped bilayer 30˚C**	0.18 (0.02)	0.17 (0.01)
**IL-doped bilayer 40˚C**	0.19 (0.03)	0.17 (0.01)

Table S1 – Bending modulus, κ, for neat and [bmim][Cl]-doped protonated DMPC lipid vesicles in D_2O at 30 ˚C and 40 ˚C obtained with the two different fitting protocols described in the *Materials and Methods* section. The uncertainties, reported in brackets, are standard deviations.

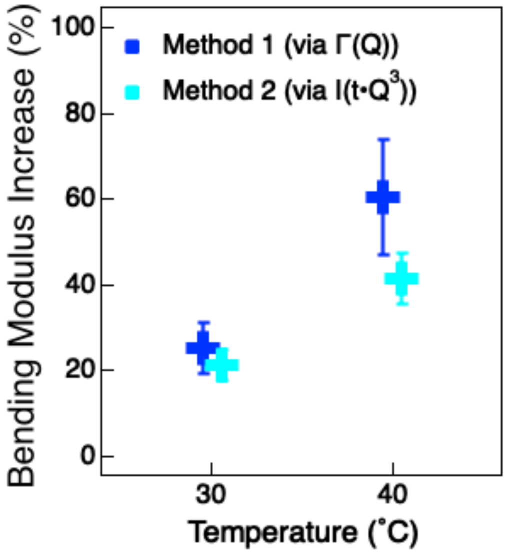

Figure S7 – Bending modulus percentage increases of IL-doped DMPC-lipid vesicles with respect to neat, $100(\kappa_{IL}-\kappa_{neat})/\kappa_{neat}$, at 30˚C and 40˚C obtained with the two fitting methods described in the *Materials and Methods* section. The data are clearly showing the IL-induced increase in bilayer bending modulus. Error bars have been calculated by directly propagating the standard deviations in κ.

<u>*Note:*</u>
Certain trade names and company products are identified in order to specify adequately the experimental procedure. In no case does such identification imply recommendation or endorsement by the authors and the National Institute of Standards and Technology, nor does it imply that the products are necessarily the best for the purpose.
